# National and state-level variations in caregiving for persons living with dementia in the US

**DOI:** 10.3389/fpubh.2026.1751603

**Published:** 2026-01-22

**Authors:** Joseph E. Gaugler, Elma Johnson, Benjamin Denno, Matthew Baumgart, Lisa C. McGuire

**Affiliations:** 1 BOLD Public Health Center of Excellence on Dementia Caregiving, School of Public Health, University of Minnesota, Minneapolis, MN, United States; 2 The Alzheimer’s Association, Chicago, IL, United States; 3 Gerontological Society of America, Washington, DC, United States

**Keywords:** Alzheimer’s disease, behavioral risk factor surveillance survey, dementia, family caregiving, informal caregiving

## Abstract

**Purpose:**

The objective of this study was to examine variations between dementia caregivers, caregivers of people without dementia, and non-caregivers across a range of sociodemographic and health variables nationally in 47 states and within five specific states representing a region of the U. S. (New York, Arizona, Minnesota, Idaho, Maine).

**Methods:**

This cross-sectional, observational study utilized 2021–2022 data from the Centers for Disease Control and Prevention (CDC) Behavioral Risk Factor Surveillance System (BRFSS). Bivariate and cross-tabulation analyses were conducted to examine empirical variation.

**Results:**

Although national results remain fairly consistent with prior research on dementia caregivers, heterogeneity emerges when comparing national results with dementia caregiving data across the five selected states.

**Conclusion:**

The availability of local data resources on dementia caregiving could help to provide more accurate/relevant estimates of ADRD caregiving prevalence and better deliver home and community-based services where they are most needed.

## 1 Introduction

Alzheimer’s disease is a public health crisis in the U. S. Over 7 million individuals in the U. S. are living with Alzheimer’s disease or a related dementia (ADRD), with nearly 12 million unpaid family members, friends, and others who provide care to people living with AD/ADRD (hereafter referred to as “caregivers”) (1). The objective of this study was to use 2021–2022 data from the nationally representative Behavioral Risk Factor Surveillance System to examine differences between dementia caregivers, caregivers of people without dementia, and non-caregivers across sociodemographic and health variables. A contribution of this analysis is to examine whether national patterns among dementia caregivers, caregivers of people without dementia, and non-caregivers differ across states within regions of the U. S.

### 1.1 Background

Despite extensive national-level research, little is known about how dementia caregiving experiences vary across U. S. states and regions. A considerable body of literature exists on the potential health consequences of dementia care. When compared to age-matched non-caregivers as well as caregivers of people without dementia, dementia caregivers report significantly greater emotional distress, care demands, financial strain, work disruptions, negative mental health outcomes, and greater healthcare service utilization (1–6). Key subgroups of dementia caregivers at particular risk for distress are consistently identified (e.g., women, spousal caregivers, caregivers of persons who exhibit multiple disruptive behaviors). However, whether and how geographical location is associated with the wellbeing of dementia caregivers remains a public health data gap, affecting the targeting and delivery of support services. Although multiple national representative data sources exist to describe dementia caregivers (7), these surveys do not yield sufficient sample sizes within states and local jurisdictions to better understand the local dynamics of dementia caregiving (e.g., dementia caregiving prevalence and health challenges at the state, county, and/or community level). The availability of local data resources on dementia caregiving could yield more accurate/relevant estimates of ADRD caregiving prevalence and identify distressed caregivers’ locations, enabling better delivery of home- and community-based services where they are most needed (8, 9). Although more local/granular data sources would be ideal to identify those communities most in need of dementia care services/supports, even state-level data could provide more refined information to, for example, update state dementia plans to better meet the needs of those living with dementia or those caring for them in a particular state.

Given that over 83% of older adults with dementia solely rely on caregivers for the help they require (10), dementia caregiving, in particular, has public health relevance. Multiple evidence-based approaches and programs exist that can alleviate distress, improve the wellbeing of dementia caregivers, and reduce service use on the part of people living with dementia (11–13). The role of public health in measuring the prevalence of dementia caregivers, in addition to the potential health characteristics of those providing dementia care, represents a public health gap, as both national and local analyses of dementia caregiving can inform the need for services, policies, and outreach for communities and families.

The goal of the current study was to provide a public health “snapshot” of dementia caregiving in the U. S. Leveraging complete, up-to-date (2021/2022), cross-sectional, representative data from a regularly administered health survey (the Behavioral Risk Factor Surveillance Survey/BRFSS), we aimed to describe and compare the health implications of dementia caregiving both nationally and on a selected state-by-state basis. Among the advantages of BRFSS are its national and state-level representation in the U. S., which enhances its public health relevance for monitoring chronic conditions and caregiving. The BRFSS is also administered annually, which lends the data recency compared to other national data sources on dementia and dementia caregiving. The use of post-pandemic data, comparisons of dementia, non-dementia, and non-caregivers, and the inclusion of cross-state as well as national analyses are also novel elements. In addition, highlighting potential inequalities in dementia caregiving health across states could help tailor state-level dementia plans to address caregiver needs in specific regions, thereby better informing public health decision-making and actions.

## 2 Methods

### 2.1 Data source and sample

We used a cross-sectional, observational design in the current study, which relied on data from the Centers for Disease Control and Prevention (CDC) Behavioral Risk Factor Surveillance System (BRFSS) (14). The BRFSS is a state-based survey that is nationally representative and administered over the telephone (landline or cell phone). Random-digit dialing is used, and participants complete surveys that assess the health of non-institutionalized adults in the United States across various domains. Participants are sampled across all 50 states, Washington, DC, and a U. S. territory. The survey collects information from over 400,000 respondents per year (14). Participants in the core survey report demographic information, health conditions, health-related behaviors, and, in an additional optional module, caregiving (among other areas). The data are publicly available and de-identified to facilitate analysis and use by researchers, public health agencies, and others for studying and understanding the nation’s public health. In this analysis, we utilize the 2021–2022 BRFSS data from states (the caregiving optional module was not administered in Florida, Montana, or Tennessee during 2021–22) that included caregiver data, providing the most comprehensive and comparable up-to-date information on dementia caregiving across states to maximize statistical power (2021 *N* = 188,494; 2022 *N* = 110,905) (14, 15). The BRFSS 2021–2022 cycles surveyed U. S. adults aged 18 and older who had landline or cellular phones in all 50 states, the District of Columbia, and U. S. territories. Adults living in institutional settings (e.g., nursing homes) were excluded. The three states that did not select to administer the Optional Caregiving BRFSS module in 2021 or 2022 were also excluded.

### 2.2 Ethics statement

The BRFSS data used for this study are de-identified and publicly available. For these reasons, the study is exempt from institutional review board review. This study also adhered to relevant ethical standards of secondary data analysis.

### 2.3 Measures

Respondents self-identified their *caregiving status* by indicating whether they provided regular care or assistance to a family member or friend with a health problem/disability in the past 30 days. Self-identified caregivers were then asked to identify the main health problem, long-term illness, or disability the care recipient lives with, choosing from a list of 15 options, including “Alzheimer’s disease, dementia, and other cognitive impairment disorders.” If respondents cared for those with multiple conditions, the “main” condition reported was used to categorize caregivers as dementia or a non-dementia caregiver (*dementia* vs. *non-dementia caregiver*). Additional relevant BRFSS data utilized for this analysis included *demographic* variables (e.g., age, race/ethnicity, income). *Caregiving context* includes the type of care provided (help managing personal care for the care recipient; help managing household tasks for the care recipient); the caregiver’s relationship to the care recipient; length of care (less than 5 years; 5 years or more); and hours of care provided in a typical week (less than 20 h per week; 20–39 h per week; 40 or more hours per week). Respondents were also asked, “Has a doctor, nurse, or other health professional ever told you that you have” one of the following *chronic conditions* (yes/no): coronary heart disease (including heart attack); stroke; cardiovascular heart disease; asthma; cancer; pulmonary disease; arthritis; diabetes; and/or hypertension (2021 BRFSS only). A summary variable was also created to indicate whether respondents reported at least one of these chronic conditions (except hypertension, which was included in the 2021 BRFSS only). To assess *healthy behaviors*, respondents were asked, “Do you now smoke cigarettes every day, some days, or not at all? (smoking).” To capture binge drinking, two items were administered: “During the past 30 days, how many days per week or per month did you have at least one drink of any alcoholic beverage such as beer, wine, a malt beverage or liquor?” and “One drink is equivalent to a 12-ounce beer, a 5-ounce glass of wine, or a drink with one shot of liquor. During the past 30 days, on the days when you drank, about how many drinks did you drink on the average?” Heavy drinkers, using BRFSS guidance (14), were operationally defined as males reporting five or more drinks and females having four or more drinks on a single occasion (yes/no). Exercise was assessed via the question, “During the past month, other than your regular job, did you participate in any physical activities or exercises such as running, calisthenics, golf, gardening, or walking for exercise?” (yes/no).

### 2.4 Statistical analysis

The prevalence of various chronic conditions, socio-demographic traits, and caregiver traits was estimated via weighted survey cross-tabulations in Stata Version 17. The analyses relied on the complex sampling weighting methodology outlined by the Centers for Disease Control and Prevention for the 2022 module data (16). In the bivariate analyses, estimates for these conditions and traits were generated for dementia caregivers, caregivers of people without dementia, and non-caregivers. Analyses were conducted with the “national” (47-state) BRFSS caregiving data and then repeated in select U. S. states. Five states were selected to represent different regions of the U. S. to ensure representation (New York, Arizona, Minnesota, Idaho, and Maine). Additionally, they reported relatively large caregiving sample sizes, enabling two-way comparisons. Per BRFSS guidelines, estimates were censored from the final results if the sample size of the observed group was below 50 or the relative standard error exceeded 30% (which occurred occasionally in this analysis when examining minority race/ethnicity populations). The statistical significance of key descriptive comparisons was approximated by examining the 95% confidence intervals of each estimate; when the confidence intervals overlapped, the disparity in estimates was most likely not statistically significant. Given the large sample sizes in both national and state descriptive analyses (which increase the risk of Type I error), we used a conservative criterion of 95% confidence interval overlap for statistical significance (17, 18).

## 3 Results

### 3.1 National findings

Table 1 presents the univariate characteristics and bivariate comparisons of the national BRFSS dementia caregiver data. Among non-dementia caregivers, the most common major health problem or illness of care recipients, as reported by caregivers, was “old age” (25.7%), an “other” reason not provided (12.9%), injuries (5.6%), heart disease (7.7%), cancer (7.3%), and diabetes (5.2%).

**Table 1:** National comparisons: caregivers of persons with dementia, persons without dementia, and non-caregivers.

Variable		47 states		Years: 2021–2022	
		Dementia caregiver (*n =* 13,565; 2021 *n =* 8,331; 2022 *n =* 5,234) 4.26% (4.08–4.45)	Non-dementia caregiver (*n* = 47,611; 2021 *n* = 30,114; 2022 *n* = 17,497) 15.75% (15.40–16.10)	Non-caregiver (*n* = 238,223; 2021 *n* = 150,049; 2022 *n* = 88, 174) 79.99% (79.60–80.37)	All surveyed (*n* = 299,399; 2021 *n* = 188,494; 2022 *n* = 88,174) 100% n/a
Sociodemographics					
Race/ethnicity	White, Non-Hisp.	69.62% (67.27–71.88)	66.75% (65.51–67.96)	62.07% (61.53–62.61)	63.13% (62.66–63.60)
	Black, Non-Hisp.	11.24% (10.06–12.54)	12.29% (11.59–13.04)	10.77% (10.47–11.08)	11.03% (10.76–11.31)
	Asian, Non-Hisp.	4.08% (2.67–6.20)	3.80% (3.16–4.57)	6.46% (6.04–6.92)	5.94% (5.58–6.33)
	American Indian/Alaska Native, Non-Hispanic	1.31% (0.99–1.73)	1.29% (1.11–1.49)	0.98% (0.9–1.07)	1.04% (0.97–1.12)
	Hispanic	10.97% (9.30–12.90)	12.50% (11.52–13.56)	17.13% (16.67–17.60)	16.14% (15.74–16.55)
	Other Race, Non-Hisp.	2.78% (2.35–3.28)	3.36% (2.98–3.79)	2.58% (2.45–2.72)	2.71% (2.59–2.84)
	<45	27.35% (25.29–29.50)	39.49% (38.20–40.80)	46.48% (45.95–47.01)	44.56% (44.08–45.04)
Age	45–64	45.05% (42.86–47.25)	37.32% (36.18–38.47)	30.11% (29.62–30.59)	31.88% (31.44–32.32)
	65 +	27.60% (25.68–29.61)	23.19% (22.32–24.08)	23.42% (23.01–23.82)	23.56% (23.20–23.92)
Income (annual)	< $50 k	42.43% (40.11–44.79)	44.23% (42.89–45.59)	42.06% (41.47–42.65)	42.43% (41.90–42.95)
	$50–99.99 k	31.26% (29.09–33.51)	30.81% (29.60–32.05)	29.19% (28.65–29.72)	29.54% (29.06–30.02)
	$100 k +	26.31% (24.06–28.69)	24.95% (23.62–26.34)	28.76% (28.21–29.31)	28.03% (27.54–28.53)
Caregiving context					
Type of care provided	Provides help managing personal care	65.78% (63.66–67.84)	45.11% (43.89–46.35)		
	Provides help manging household tasks	84.64% (82.94–86.19)	77.02% (75.93–78.07)		
Care recipient’s relationship to caregiver	Spouse/partner	16.49% (14.75–18.40)	22.27% (21.14–23.44)		
	Parent/parent-in-Law	45.09% (42.82–47.38)	31.73% (30.55–32.95)		
	Other	38.42% (36.09–40.79)	46.00% (44.58–47.42)		
Length of care	Less than 5 years	67.95% (65.85–69.97)	69.23% (68.13–70.31)		
	5 years or more	32.05% (30.03–34.15)	30.77% (29.69–31.87)		
Hours of care	Less than 20 h per week	58.78% (56.53–60.99)	70.92% (69.78–72.05)		
	20–39 h per week	14.00% (12.47–15.69)	10.68% (10.01–11.38)		
	40 + h per week	27.22% (25.30–29.23)	18.40% (17.42–19.41)		
Chronic conditions					
Percent reporting	Coronary heart disease (including heart attack)	8.57% (7.42–9.87)	7.16% (6.69–7.67)	6.40% (6.16–6.65)	6.62% (6.40–6.84)
	Stroke	4.08% (3.40–4.88)	3.77% (3.36–4.24)	3.33% (3.16–3.51)	3.43% (3.27–3.59)
	Cardiovascular disease (CHD or stroke)	11.09% (9.82–12.50)	9.84% (9.23–10.48)	8.64% (8.37–8.93)	8.93% (8.69–9.19)
	Asthma	18.77% (17.13–20.54)	18.42% (17.51–19.36)	14.24% (13.87–14.61)	15.09% (14.75–15.43)
	Cancer	13.25% (11.47–15.26)	10.15% (9.40–10.94)	8.24% (7.93–8.56)	8.76% (8.47–9.05)
	Pulmonary disease	10.82% (9.53–12.27)	8.52% (7.96–9.11)	6.24% (6.01–6.47)	6.79% (6.58–7.01)
	Arthritis	42.35% (39.87–44.88)	37.48% (36.21–38.77)	27.29% (26.77–27.82)	29.56% (29.08–30.04)
	Diabetes	13.32% (12.02–14.74)	12.68% (12.03–13.37)	11.66% (11.32–12.00)	11.89% (11.60–12.19)
	Hypertension (2021 Only)	42.24% (39.27–45.26)	36.92% (35.43–38.43)	32.06% (31.43–32.68)	33.25% (32.68–33.82)	
At least one of the above chronic conditions (excludes hypertension)	46.83% (44.65–49.01)	42.50% (41.33–43.67)	35.45% (34.96–35.94)	37.04% (36.60–37.48)
Health behaviors					
Smoking	Current smoker	16.62% (15.20–18.13)	16.78%(15.98– 17.62)	11.80% (11.48–12.13)	12.79% (12.50–13.09)
Alcohol	Binge drinker	15.13% (13.21–17.27)	15.52% (14.49–16.60)	16.39% (15.94–16.85)	16.19% (15.79–16.61)
Physical activity	No physical activity (past 30 Days, outside of employment)	21.5% (19.9–23.1)	21.9% (20.9–22.8)	24.2% (23.7–24.6)	23.7% (23.3–24.1)

Excludes Florida, Montana, and Tennessee and does not include District of Columbia; “Dementia Caregivers” as indicated here refers to caregivers who either identify dementia as the “main condition” of their care recipient; Under each statistic is the 95% confidence interval; “--” Indicates that the result is suppressed, due to relative standard error > 30%.

#### 3.1.1 Race/ethnicity

Over six in 10 respondents to the 2021–2022 BRFSS were White/Non-Hispanic. Compared with non-caregivers, dementia caregivers were more likely to be White/Non-Hispanic (62.07% vs. 69.62%, respectively). In contrast, non-dementia caregivers were more likely to be Hispanic when compared to dementia caregivers (17% vs. 11%, respectively).

#### 3.1.2 Income

Dementia caregivers did not differ significantly from other caregivers in their reported annual income.

#### 3.1.3 Type of care provided

Dementia caregivers were significantly more likely to assist care recipients in managing personal care and household tasks than caregivers of people without dementia (66% vs. 45%; 85% vs. 77%, respectively).

#### 3.1.4 Length of care

No significant differences in the length of care were observed between caregivers of people with and without dementia.

#### 3.1.5 Chronic conditions

Dementia caregivers were more likely than non-caregivers to report several chronic conditions, including coronary heart disease (8.57% vs. 6.40%), cardiovascular disease (11.09% vs. 8.64%), asthma (18.77% vs. 14.24%), and diabetes (13.32% vs. 11.66%). In addition, dementia caregivers were more likely to report cancer (13.25%), pulmonary disease (10.82%), arthritis (42.35%) and hypertension (42.24%) than caregivers of people without dementia (10.15, 8.52, 37.48, 36.92%, respectively) and non-caregivers (8.24, 6.24, 27.29, 11.66, 32.06%, respectively). Dementia caregivers were also more likely to indicate at least one chronic condition (46.83%) when compared to caregivers of people without dementia (42.50%) and non-caregivers (35.45%).

#### 3.1.6 Health behaviors

Compared to non-caregivers, dementia caregivers were more likely to smoke (12 to 17%, respectively). However, 24% of non-caregivers were more likely to indicate no physical activity in the past 30 days outside of employment than the 22% of dementia caregivers.

#### 3.1.7 Care recipient relationship

Caregivers of people without dementia were more likely to be spousal caregivers than dementia caregivers (19% vs. 14%, respectively); in contrast, dementia caregivers were more likely to be adult children than caregivers of people without dementia (45% vs. 33%).

#### 3.1.8 Hours of care

Dementia caregivers were more likely to provide over 40 h per week of care to care recipients (27%) than caregivers of people without dementia (18%).

#### 3.1.9 Age

Dementia caregivers, compared with non-dementia caregivers, were significantly less likely to be under 45 (27.4% v. 39.5%) and more likely to be middle-aged (45.1% v. 37.3%) or 65 and older (27.6% v. 23.2%).

### 3.2 State-level findings

Tables 2–6 provide univariate characteristics and bivariate comparisons of the state-level BRFSS dementia caregiver data.

**Table 2:** Bivariate comparisons, New York.

NY					
Variable		Dementia caregiver (*n* = 446) 3.41% (2.91–4.00)	Non-dementia caregiver (*n* = 1,705) 14.82% (13.62–16.10)	Non-caregiver (*n* = 8,466) 81.77% (80.42–83.04)	All surveyed (*n* = 10,617) 100%
Sociodemographics					
Race/ethnicity	White, Non-Hisp.	59.71% (51.24–67.63)	69.22% (65.11–73.05)	55.65% (53.95–57.34)	57.80% (53.95–57.34)
	Black, Non-Hisp.	13.59% (8.64–20.72)	11.46% (9.10–14.33)	13.80% (12.52–15.18)	13.44% (12.32–14.65)
	Asian, Non-Hisp.	–	3.94% (2.29–6.72)	7.91% (6.77–9.23)	7.24% (6.25–8.37)
	American Indian/Alaska Native, Non-H.	–	–	1.49% (0.98–2.25)	1.46% (1.02–2.08)
	Hispanic	16.19% (10.20–24.71)	12.18% (9.82–15.02)	19.30% (17.91–20.77)	18.14% (16.93–19.42)
	Other Race, Non-Hisp.	–	2.15% (1.22–3.74)	1.85% (1.55–2.22)	1.92% (1.62–2.27)
Income (annual)	< $50 k	44.32% (35.81–53.17)	44.96% (40.15–49.88)	43.70% (41.69–45.72)	43.91% (42.11–45.73)
	$50–99.99 k	28.46% (20.85–37.54)	26.75% (22.82–31.08)	27.69% (25.91–29.54)	27.57% (25.97–29.23)
	$100 k +	27.22% (20.39–35.32)	28.29% (23.74–33.32)	28.62% (26.75–30.57)	28.52% (26.84–30.27)
Caregiving context					
Type of care provided	Provides help managing personal care	67.74% (59.93–74.67)	38.17% (33.75–42.80)		
	Provides help manging household tasks	84.56% (78.12–89.37)	73.77% (69.56–77.59)		
Care recipient’s relationship to caregiver	Spouse/partner	10.05% (6.71–14.79)	11.76% (9.60–14.33)		
	Parent/parent-in-Law	39.85% (32.31–47.91)	37.00% (32.81–41.39)		
	Other	50.10% (42.08–58.11)	51.25% (46.68–55.79)		
Length of care	Less than 5 years	64.84% (56.91–72.03)	71.36% (67.37–75.04)		
	5 years or more	35.16% (27.97–43.09)	28.64% (24.96–32.63)		
Hours of care	Less than 20 h per week	56.16% (47.98–64.02)	73.73% (69.70–77.40)		
	20–39 h per week	16.79% (11.17–24.44)	9.93% (7.64–12.80)		
	40 + hours per week	27.06% (20.46–34.84)	16.34% (13.46–19.70)		
Chronic conditions					
Percent reporting	Coronary heart disease (including heart attack)	–	6.16% (4.56–8.28)	6.81% (6.01–7.71)	6.74% (6.02–7.55)
	Stroke	–	3.06% (2.02–4.59)	2.49% (2.03–3.05)	2.64% (2.20–3.16)

	Cardiovascular disease (CHD or Stroke)	–	8.05% (6.24–10.33)	8.36% (7.49–9.33)	8.40% (7.61–9.28)
	Asthma	17.00% (11.67–24.10)	17.18% (14.43–20.33)	13.77% (12.57–15.07)	14.39% (13.29–15.56)
	Cancer	9.94% (6.12–15.75)	6.82% (5.36–8.65)	7.30% (6.53–8.15)	7.32% (6.62–8.08)
	Pulmonary disease	9.02% (5.16–15.29)	7.68% (5.99–9.80)	6.24% (5.43–7.15)	6.55% (5.82–7.36)
	Arthritis	38.17% (30.86–46.05)	30.76% (27.23–34.52)	23.94% (22.54–25.39)	25.43% (24.15–26.76)
	Diabetes	14.35% (9.71–20.69)	12.92% (10.48–15.83)	11.88% (10.77–13.10)	12.12% (11.11–13.21)
	Hypertension (2021 Only)	37.59% (30.26–45.54)	32.45% (28.79–36.35)	31.32% (29.72–32.96)	31.70% (30.26–33.18)
At least one of the above chronic conditions (excludes hypertension)	59.52% (51.50–67.07)	50.16% (45.61–54.70)	44.55% (42.79–46.33)	45.89% (44.28–47.52)
Health behaviors					
Smoking	Current smoker	15.39% (10.40–22.17)	17.81% (14.42–21.81)	11.65% (10.57–12.82)	12.69% (11.64–13.83)
Alcohol	Binge drinker	13.38% (8.25–20.98)	15.58% (12.24–19.62)	14.59% (13.30–15.99)	14.69% (13.49–15.98)
Physical activity	No physical activity (past 30 Days, outside of employment)	20.24% (14.81–27.03)	21.32% (17.94–25.15)	26.48% (24.91–28.12)	25.50% (24.10–26.96)

**Table 3:** Bivariate comparisons, Arizona.

AZ					
Variable		Dementia caregiver (*n* = 217) 4.99% (4.10–6.06)	Non-dementia caregiver (*n* = 733) 16.48% (14.69–18.44)	Non-caregiver (*n* = 3,013) 78.53% (76.42–80.49)	All surveyed (*n* = 3,963) 100%
Sociodemographics					
Race/ethnicity	White, Non-Hisp.	69.70% (59.23–78.45)	63.90% (57.43–69.90)	57.13% (54.14–60.06)	58.87% (56.25–61.44)
	Black, Non-Hisp.	–	7.04% (3.73–12.91)	3.59% (2.58–4.99)	4.28% (3.22–5.68)
	Asian, Non-Hisp.	–	–	4.36% (3.03–6.23)	3.71% (2.62–5.23)
	American Indian/Alaska Native, Non-H.	–	3.42% (2.06–5.64)	3.58% (2.68–4.77)	3.42% (2.66–4.39)
	Hispanic	18.07% (11.16–27.91)	21.60% (16.84–27.27)	27.95% (25.20–30.88)	26.41% (24.05–28.91)
	Other Race, Non-Hisp.	–	–	3.39% (2.44–4.69)	3.30% (2.45–4.43)
Income (annual)	< $50 k	46.25% (35.94–56.89)	45.66% (39.10–52.38)	43.52% (40.44–46.65)	44.05% (41.35–46.78)
	$50–99.99 k	30.45% (21.72–40.86)	37.30% (31.08–43.96)	30.95% (28.08–33.97)	32.03% (29.49–34.68)
	$100 k +	23.30% (15.38–33.67)	17.04% (12.77–22.38)	25.53% (22.89–28.36)	23.92% (21.67–26.33)
Caregiving context					
Type of care provided	Provides help managing personal care	62.35% (52.21–71.52)	42.95% (36.76–49.36)		
	Provides help manging household tasks	80.56% (71.40–87.31)	78.06% (73.05–82.37)		
Care recipient’s relationship to caregiver	Spouse/partner	16.70% (11.10–24.35)	20.72% (16.21–26.10)		
	Parent/parent-in-law	42.84% (33.50–52.94)	32.71% (26.79–39.23)		
	Other	40.46% (30.92–50.78)	46.57% (40.37–52.88)		
Length of care	Less than 5 years	66.50% (56.09–75.72)	70.67% (64.89–75.85)		
	5 years or more	33.50% (24.48–43.91)	29.33% (24.15–35.11)		
Hours of care	Less than 20 h per Week	59.83% (49.30–69.54)	67.80% (60.81–74.07)		
	20–39 h per Week	11.58% (7.04–18.45)	14.44% (9.80–20.76)		
	40 + h per Week	28.59% (19.91–39.19)	17.77% (12.98–23.84)		
Chronic conditions					
Percent reporting	Coronary heart disease (including heart attack)	11.70% (7.00–18.91)	11.56% (7.67–17.07)	6.91% (5.84–8.17)	7.91% (6.76–9.24)
	Stroke	–	–	4.49% (3.45–5.82)	4.58% (3.63–5.76)
	Cardiovascular disease (CHD or stroke)	11.92% (7.18–19.15)	14.00% (9.87–19.49)	10.08% (8.64–11.73)	10.81% (9.42–12.38)
	Asthma	17.00% (10.83–25.66)	20.54% (15.63–26.51)	15.84% (13.75–18.18)	16.67% (14.77–18.76)
	Cancer	10.84% (6.75–16.97)	9.05% (6.70–12.11)	8.66% (7.49–9.98)	8.83% (7.79–9.99)
	Pulmonary disease	9.81% (5.32–17.39)	7.36% (5.23–10.25)	6.84% (5.60–8.34)	7.08% (5.98–8.35)
	Arthritis	41.94% (32.65–51.85)	34.48% (29.29–40.07)	24.34% (22.20–26.62)	26.89% (24.92–28.96)
	Diabetes	18.28% (11.64–27.53)	13.34% (10.06–17.49)	12.78% (11.16–14.60)	13.15% (11.69–14.76)
	Hypertension (2021 only)	–	–	–	–
	At least one of the above chronic conditions (excludes Hypertension)	64.89% (54.77–73.82)	62.04% (55.88–67.83)	47.69% (44.81–50.58)	50.91% (48.36–53.47)
Health behaviors					
Smoking	Current smoker	13.26% (8.24–20.65)	15.59% (11.13–21.40)	11.93% (10.20–13.91)	12.59% (10.98–14.40)
Alcohol	Binge drinker	16.53% (10.07–25.96)	20.55% (15.30–27.03)	15.59% (13.40–18.07)	16.45% (14.44–18.68)
Physical activity	No physical activity (past 30 Days, outside of employment)	22.71% (15.59–31.84)	21.12% (16.60–26.47)	24.72% (22.28–27.32)	24.02% (21.91–26.27)

**Table 4:** Bivariate comparisons, Minnesota.

MN					
Variable		Dementia caregiver (*n* = 538) 3.65% (3.30–4.04)	Non-dementia caregiver (*n* = 1,788) 13.07% (12.39–13.78)	Non-caregiver (*n* = 11,251) 83.28% (82.50–84.03)	All surveyed (*n* = 13,577) 100%
Sociodemographics					
Race/ethnicity	White, Non-Hisp.	85.39% (80.96–88.93)	82.54% (79.83–84.94)	82.65% (81.71–83.56)	82.74% (81.88–83.57)
	Black, Non-Hisp.	5.07% (2.88–8.77)	6.35% (4.85–8.27)	5.32% (4.76–5.95)	5.45% (4.92–6.03)
	Asian, Non-Hisp.	–	3.18% (2.09–4.82)	4.95% (4.37–5.59)	4.62% (4.11–5.18)
	American Indian/Alaska Native, Non-H.	–	2.19% (1.32–3.60)	0.71% (0.56–0.90)	0.93% (0.75–1.16)
	Hispanic	2.66% (1.60–4.41)	3.21% (2.41–4.27)	4.92% (4.47–5.42)	4.61% (4.22–5.05)
	Other Race, Non-Hisp.	3.17% (1.79–5.56)	2.54% (1.60–3.99)	1.45% (1.24–1.69)	1.65% (1.42–1.92)
Income (annual)	< $50 k	42.07% (36.70–47.64)	39.17% (36.19–42.24)	35.17% (33.99–36.36)	35.95% (34.88–37.04)
	$50–99.99 k	31.46% (26.62–36.73)	35.42% (32.61–38.33)	33.57% (32.40–34.75)	33.73% (32.68–34.80)
	$100 k +	26.47% (22.26–31.17)	25.41% (22.88–28.11)	31.27% (30.18–32.38)	30.32% (29.34–31.32)
Caregiving context					
Type of care provided	Provides help managing personal care	59.59% (54.55–64.44)	43.86% (40.99–46.77)		
	Provides help manging household tasks	87.07% (83.19–90.16)	81.06% (78.82–83.10)		
Care recipient’s relationship to caregiver	Spouse/Partner	15.57% (12.42–19.33)	17.53% (15.62–19.61)		
	Parent/Parent-in-Law	46.08% (41.00–51.24)	33.66% (30.97–36.45)		
	Other	38.36% (33.42–43.54)	48.82% (45.93–51.71)		
Length of care	Less than 5 years	67.35% (62.31–72.01)	69.33% (66.58–71.96)		
	5 years or more	32.65% (27.99–37.69)	30.67% (28.04–33.42)		
Hours of care	Less than 20 h per week	65.41% (60.29–70.19)	74.09% (71.34–76.67)		
	20–39 h per week	12.31% (9.35–16.04)	11.13% (9.32–13.23)		
	40 + hours per week	22.28% (18.20–26.98)	14.78% (12.75–17.07)		
Chronic conditions					
Percent reporting	Coronary heart disease (including heart attack)	6.95% (4.84–9.89)	5.98% (4.85–7.34)	5.29% (4.86–5.77)	5.44% (5.04–5.88)
	Stroke	2.72% (1.53–4.78)	1.87% (1.30–2.68)	2.66% (2.31–2.68)	2.56% (2.25–2.91)
	Cardiovascular Disease (CHD or Stroke)	8.57% (6.26–11.62)	7.48% (6.21–8.98)	7.22% (6.68–7.79)	7.30% (6.81–7.82)
	Asthma	17.75% (14.07–22.15)	15.51% (13.55–17.69)	12.34% (11.61–13.11)	12.95% (12.27–13.67)
	Cancer	11.18% (8.32–14.86)	7.53% (6.31–8.97)	7.38% (6.86–7.93)	7.54% (7.06–8.05)
	Pulmonary Disease	6.55% (4.26–9.93)	5.45% (4.34–6.83)	4.60% (4.14–5.10)	4.78% (4.36–5.24)
	Arthritis	32.42% (27.89–37.31)	28.25% (25.90–30.72)	21.96% (21.09–22.86)	23.16% (22.36–23.99)
	Diabetes	9.07% (6.73–12.11)	10.88% (9.36–12.61)	8.92% (8.33–9.55)	9.18% (8.64–9.76)
	Hypertension (2021 Only)	35.00% (30.38–39.92)	33.02% (30.48–35.66)	29.20% (28.23–30.20)	29.91% (29.02–30.83)
	At Least One of the Above Chronic Conditions (excludes Hypertension)	54.04% (48.89–59.11)	47.44% (44.62–50.28)	41.38% (40.29–42.49)	42.64% (41.63–43.64)
Health behaviors					
Smoking	Current smoker	16.67% (13.12–20.97)	18.42% (16.14–20.94)	12.43% (11.67–13.23)	13.37% (12.65–14.12)
Alcohol	Binge drinker	14.24% (11.07–18.15)	17.24% (15.00–19.75)	17.88% (17.01–18.78)	17.66% (16.87–18.48)
Physical activity	No physical activity (past 30 Days, outside of employment)	17.64% (14.04–21.91)	19.43% (17.27–21.79)	20.37% (19.45–21.33)	20.15% (19.32–21.01)

**Table 5:** Bivariate comparisons, Idaho.

ID					
Variable		Dementia caregiver (*n* = 282) 4.32% (3.74–4.98)	Non-dementia caregiver (*n* = 976) 14.70% (13.67–15.80)	Non-caregiver (*n* = 4,853) 80.98% (79.77–82.14)	All surveyed (*n* = 6,111) 100%
Sociodemographics					
Race/ethnicity	White, Non-Hisp.	89.17% (83.18–93.20)	88.52% (85.53–90.95)	83.43% (81.90–84.86)	84.43% (83.11–85.66)
	Black, Non-Hisp.	–	–	0.78% (0.48–1.25)	0.77% (0.51–1.17)
	Asian, Non-Hisp.	–	–	0.80% (0.49–1.30)	0.79% (0.51–1.21)
	American Indian/Alaska Native, Non-H.	–	–	1.31% (0.93–1.84)	1.35% (1.00–1.82)
	Hispanic	–	6.53% (4.65–9.10)	11.39% (10.16–12.75)	10.42% (9.36–11.58)
	Other Race, Non-Hisp.	–	2.08% (1.22–3.55)	2.28% (1.79–2.91)	2.24% (1.81–2.78)
Income (annual)	<$50 k	56.42% (48.53–63.99)	52.52% (48.28–56.73)	45.63% (43.74–47.52)	47.15% (45.48–48.84)
	$50–99.99 k	28.24% (21.70–35.86)	28.43% (24.73–32.43)	32.32% (30.53–34.16)	31.55% (29.97–33.18)
	$100 k +	15.34% (10.74–21.43)	19.05% (15.81–22.78)	22.05% (20.47–23.73)	21.30% (19.90–22.76)
Caregiving context					
Type of care provided	Provides help managing personal care	56.67% (49.14–63.90)	45.16% (41.29–49.09)		
	Provides help manging household tasks	87.54% (82.28–91.41)	81.70% (78.50–84.51)		
Care recipient’s relationship to caregiver	Spouse/partner	16.18% (11.85–21.69)	21.18% (18.41–24.24)		
	Parent/parent-in-law	41.86% (34.93–49.12)	27.44% (24.08–31.07)		
	Other	41.97% (34.66–49.64)	51.38% (47.48–55.27)		
Length of care	Less than 5 years	64.63% (57.44–71.22)	66.37% (62.52–70.02)		
	5 years or more	35.37% (28.78–42.56)	33.63% (29.98–37.48)		
Hours of care	Less than 20 h per week	59.20% (51.83–66.18)	72.17% (68.34–75.70)		
	20–39 h per week	13.77% (9.51–19.51)	9.63% (7.43–12.41)		
	40 + hours per week	27.03% (21.14–33.87)	18.20% (15.30–21.51)		
Chronic conditions					
Percent reporting	Coronary heart disease (including heart attack)	10.18% (6.92–14.73)	7.29% (5.65–9.35)	5.51% (4.84–6.26)	5.97% (5.35–6.66)
	Stroke	–	3.16% (2.04–4.86)	2.67% (2.22–3.21)	2.82% (2.39–3.32)
	Cardiovascular disease (CHD or Stroke)	13.04% (9.15–18.24)	9.30% (7.37–11.65)	7.49% (6.71–8.36)	8.00% (7.27–8.79)
	Asthma	14.10% (10.21–19.16)	18.70% (15.75–22.05)	13.32% (12.15–14.59)	14.15% (13.07–15.29)
	Cancer	11.96% (8.40–16.74)	10.81% (8.71–13.34)	6.64% (5.93–7.43)	7.48% (6.80–8.23)
	Pulmonary disease	10.18% (6.73–15.11)	7.06% (5.38–9.21)	5.41% (4.69–6.24)	5.86% (5.19–6.61)
	Arthritis	39.41% (32.70–46.54)	33.25% (29.74–36.95)	23.66% (22.31–25.07)	25.75% (24.49–27.05)
	Diabetes	12.42% (8.61–17.59)	11.32% (9.28–13.73)	9.52% (8.64–10.49)	9.91% (9.10–10.78)
	Hypertension (2021 Only)	43.02% (36.06–50.27)	34.79% (31.34–38.40)	29.90% (28.41–31.44)	31.19% (29.84–32.57)
	At least one of the above chronic conditions (excludes hypertension)	59.66% (52.05–66.83)	56.65% (52.77–60.45)	42.61% (40.91–44.32)	45.41% (43.87–46.95)
Health behaviors					
Smoking	Current smoker	15.32% (10.95–21.03)	18.44% (15.40–21.92)	12.46% (11.34–13.68)	13.46% (12.41–14.59)
Alcohol	Binge drinker	11.50% (7.53–17.18)	11.25% (8.86–14.19)	14.61% (13.32–15.99)	13.97% (12.85–15.18)
Physical activity	No physical activity (past 30 Days, outside of employment)	16.87% (12.29–22.70)	18.21% (15.48–21.30)	21.13% (19.79–22.54)	20.52% (19.34–21.75)

**Table 6:** Bivariate comparisons, Maine.

ME					
Variable		Dementia caregiver (*n* = 239) 4.38% (3.68–5.21)	Non-dementia caregiver (*n* = 763) 13.58% (12.33–14.95)	Non-caregiver (*n* = 4,325) 82.03% (80.52–83.45)	All surveyed (*n* = 5,327) 100%
Sociodemographics					
Race/ethnicity	White, Non-Hisp.	92.68% (85.73–96.39)	94.95% (91.66–96.98)	93.75% (92.47–94.83)	93.87% (92.73–94.84)
	Black, Non-Hisp.	–	–	0.92% (0.50–1.68)	0.91% (0.52–1.57)
	Asian, Non-Hisp.	–	–	–	0.25% (0.14–0.45)
	American Indian/Alaska Native, Non-H.	–	–	0.62% (0.39–0.99)	0.72% (0.48–1.07)
	Hispanic	–	–	1.86% (1.32–2.61)	1.71% (1.25–2.34)
	Other Race, Non-Hisp.	–	–	2.56% (1.87–3.49)	2.55% (1.91–3.39)
Income (annual)	<$50 k	47.43% (37.69–57.36)	46.83% (41.02–52.72)	43.13% (40.71–45.57)	43.84% (41.66–46.04)
	$50–99.99 k	34.14% (25.19–44.38)	35.42% (30.06–41.18)	34.47% (32.12–36.89)	34.59% (32.48–36.75)
	$100 k +	18.44% (12.49–26.36)	17.75% (13.78–22.56)	22.41% (20.48–24.46)	21.57% (19.87–23.38)
Caregiving context					
Type of care provided	Provides help managing personal care	64.23% (55.73–71.92)	39.31% (34.41–44.42)		
	Provides help manging household tasks	89.65% (83.98–93.46)	75.18% (75.05–82.04)		
Care recipient’s relationship to caregiver	Spouse/partner	16.37% (11.54–22.70)	19.74% (16.24–23.78)		
	Parent/parent-in-law	44.68% (35.94–53.77)	27.65% (23.27–32.51)		
	Other	38.94% (30.15–48.52)	52.61% (47.34–57.82)		
Length of care	Less than 5 years	64.28% (55.46–72.73)	69.88% (65.08–74.28)		
	5 years or more	35.72% (27.77–44.54)	30.12% (25.72–34.92)		
Hours of care	Less than 20 h per week	56.75% (46.64–66.33)	73.53% (68.69–77.87)		
	20–39 h per week	12.49% (6.90–21.54)	9.97% (6.93–14.14)		
	40 + hours per week	30.76% (22.30–40.75)	16.50% (12.86–20.93)		
Chronic conditions					
Percent reporting	Coronary heart disease (including heart attack)	7.37% (4.10–12.90)	8.76% (6.43–11.81)	7.26% (6.36–8.28)	7.47% (6.63–8.40)
	Stroke	–	3.46% (2.11–5.63)	3.40% (2.76–4.18)	3.39% (2.81–4.08)
	Cardiovascular disease (CHD or stroke)	9.88% (6.17–15.48)	11.07% (8.43–14.40)	9.60% (8.54–10.77)	9.81% (8.84–10.88)
	Asthma	19.82% (13.40–28.32)	14.84% (11.66–18.72)	16.87% (15.19–18.70)	16.72% (15.23–18.33)
	Cancer	9.09% (5.04–15.83)	8.66% (6.48–11.47)	8.44% (7.51–9.46)	8.49% (7.64–9.44)
	Pulmonary disease	10.83% (7.01–16.36)	10.06% (7.62–13.18)	7.85% (6.87–8.95)	8.28% (7.37–9.28)
	Arthritis	38.55% (30.69–47.05)	38.74% (34.10–43.58)	29.74% (27.93–31.61)	31.35% (29.69–33.05)
	Diabetes	8.97% (5.29–14.82)	10.47% (8.15–13.37)	10.31% (9.19–11.55)	10.27% (9.27–11.37)
	Hypertension (2021 only)	34.85% (27.16–43.42)	35.64% (31.16–40.39)	32.51% (30.63–34.45)	33.04% (31.34–34.79)
	At least one of the above chronic conditions (excludes hypertension)	62.09% (52.94–70.46)	56.44% (51.10–61.64)	50.92% (48.73–53.12)	52.16% (50.19–54.13)
Health behaviors					
Smoking	Current smoker	18.68% (11.76–28.37)	21.39% (17.30–26.14)	14.18% (12.71–15.79)	15.36% (13.96–16.86)
Alcohol	Binge drinker	6.35% (3.36–11.70)	12.83% (9.47–17.15)	16.03% (14.30–17.92)	15.17% (13.66–16.82)
Physical activity	No physical activity (past 30 Days, Outside of employment)	19.98% (13.59–28.40)	22.02% (18.15–26.45)	26.72% (24.82–28.71)	25.79% (24.11–27.54)

#### 3.2.1 Race/ethnicity

In New York, fewer caregivers of people without dementia were Asian/non-Hispanic than non-caregivers (4% vs. 8%, respectively). In Minnesota, fewer dementia caregivers were Hispanic than non-caregivers (3% vs. 5%, respectively), while more dementia caregivers identified as other race, non-Hispanic than non-caregivers (3% vs. 1%, respectively). In Idaho, more caregivers of people without dementia were White, non-Hispanic than non-caregivers (89% vs. 83%, respectively), while fewer caregivers of people without dementia were Hispanic (7%) than non-caregivers (11%).

#### 3.2.2 Income

No statistically significant differences were found between dementia and caregivers of people without dementia within New York, Minnesota, Arizona, Idaho, and Maine BRFSS respondents.

#### 3.2.3 Type of care provided

In New York, Minnesota, and Maine, dementia caregivers were more likely to provide help with managing care (68, 60, 64%) and household tasks (85, 87, 90%) than caregivers of people without dementia (38% and 74%; 44% and 81%; 93%, and 75%, respectively). In Arizona and Idaho, dementia caregivers were more likely to help manage personal care tasks than caregivers of people without dementia (63% vs. 43%; 57% vs. 45%, respectively) but about equally as likely as caregivers of people without dementia to help with household tasks.

#### 3.2.4 Length of care

No significant differences were apparent in the length of care provided in any state between dementia and caregivers of people without dementia.

#### 3.2.5 Chronic conditions

In New York, Arizona, Minnesota, and Idaho, dementia caregivers were more likely to indicate arthritis (38, 42, 32, and 39%, respectively) than non-caregivers (24, 24, 22, 24, and 24%, respectively). Idaho dementia caregivers were more likely to report coronary heart disease, cardiovascular disease, cancer, pulmonary disease, and hypertension (10, 13, 12, 10, and 43%, respectively) when compared to non-caregivers (6, 7, 7, 5, and 30%, respectively). Dementia caregivers in Minnesota were also more likely to indicate asthma, cancer, and hypertension (18, 11, and 35%, respectively) than non-caregivers (12, 7, and 22%, respectively). In New York, Arizona, Minnesota, and Idaho, dementia caregivers were also more likely to indicate at least one chronic condition (60, 65, 54, and 60%, respectively) than non-caregivers (45, 48, 41, and 43%, respectively).

#### 3.2.6 Health behaviors

In New York, Minnesota, Idaho, and Maine, caregivers of people without dementia were more likely to be current smokers (18, 18, 18, and 21%, respectively) than non-caregivers (12, 12, 13, and 14%, respectively). In Maine, dementia caregivers were less likely to be binge drinkers (6%) than non-caregivers (16%).

## 4 Discussion

The objective of the current study was to describe key subgroups of dementia caregivers at the national level and to determine whether national differences persisted when considering state-level BRFSS data. The findings suggest that although national results in the 2021–2022 BRFSS data remain fairly consistent with prior research on national samples of dementia caregivers (1), heterogeneity emerges when considering and comparing national results to state-level dementia caregiving data.

In some instances, differences between national and state-level dementia caregiving data were not surprising. For example, a greater proportion of dementia caregivers in Idaho were White/Non-Hispanic (nearly 90%) when compared to national dementia caregivers (a little over 6 in 10), which is in keeping with the demographic makeup of Idaho versus the broader U. S. Similarly, in four of the five states as well as nationally, dementia caregivers were more likely to care for parents or parents-in-law than caregivers of people without dementia. When considering type of care provided, a general pattern emerged across both national and state-level dementia caregiving data, where dementia caregivers were more likely to provide household and personal care assistance than caregivers of people without dementia, although it should also be noted that such patterns were not consistent across all five states (i.e., in Arizona and Idaho dementia caregivers were more likely to provide personal care than caregivers of people without dementia; in New York, Minnesota, and Maine, the same pattern occurred but for household tasks only).

There was varying consistency across national and state findings in health behaviors and chronic conditions. Nationally and across four of the five states, dementia caregivers were more likely to be smokers than caregivers of people without dementia. Although non-caregivers appeared less physically active than dementia caregivers nationwide, these differences were not apparent at the state level. Although dementia caregivers were more likely than caregivers of people without dementia to provide more than 40 h per week of help nationally, this difference was observed only in New York and Minnesota, not in the other three states included. Nationally and in four of the five states considered, dementia caregivers were more likely to indicate at least one chronic condition or arthritis than caregivers of people without dementia. There was little consistency between state-level findings and national findings for other chronic conditions.

The findings here suggest that state-level data on dementia caregiving may inform targeted outreach, programmatic innovations, and policy recommendations that meet the diverse needs of caregivers in a given state, which may differ from those identified in national samples (9). To some degree, this work has occurred in several states in the U. S. Through the Building our Largest Data Infrastructure (BOLD) Act, which supports states in their efforts to develop and implement state dementia plans (19), The Centers for Disease Control and Prevention has created state-level infographics on caregiving, based on BRFSS data, to provide snapshots of caregiving and dementia caregiving within specific states. Of the 43 states that have received BOLD funding, several have built on these infographics to create data dashboards that demonstrate changes in key risk factors and dementia caregiving indicators over time, thereby informing local legislatures and other decision-makers and elevating dementia as a public health concern (20).

Although methodological variations persist (e.g., how caregivers are identified, which can influence estimates of caregiving prevalence in the U. S.) (7), there now exist multiple national data sources upon which to draw inferences on dementia caregiving prevalence as well as to conduct empirical comparisons between dementia caregivers, caregivers of people without dementia, and non-caregivers (matched on key characteristics such as age) to highlight the emotional, social, psychological, financial, and physical health consequences of dementia care provision across the country in general (1, 7, 21, 22). The emergence of representative national data sets has allowed researchers, providers, policymakers, and advocates to highlight the extent, costs, and health implications of dementia care. They have thus been instrumental in elevating unpaid dementia care as a public health priority. However, a key public health action to better support people living with dementia and those caring for them is to continue conducting surveillance at the state and local levels, which includes measuring the number of dementia caregivers in a given area and documenting their health risks (8, 23).

As the current study’s findings emphasize, dementia caregiver characteristics, comorbidities, and subjective health indices may vary between state-specific and national samples. Having representative data that provides greater local insights (e.g., not only at the state but also at the county, city, or town level) on dementia caregiver prevalence and wellbeing may facilitate the creation of programs or policies that best meet the needs of dementia caregivers in our local communities. Given that policy movement related to public health and dementia care is unpredictable at the federal level, engaging local or state policymakers with immediately relevant data on dementia care could lead to program and service innovations that address the localized needs of families and others who provide unpaid support to people living with dementia.

Beyond the BRFSS, several state-level efforts aim to better capture dementia prevalence, including state registries (Georgia, South Carolina, Virginia, and West Virginia all have or are establishing state dementia registries, and rely on various methodologies to do so). Broadening these efforts to capture caregivers’ availability and needs may be one strategy to better target dementia care services and support at the local level. Although it would not yield a representative sample, there may be opportunities to leverage caregiving data from the recent Centers for Medicare and Medicaid Services Guiding an Improved Dementia Experience (GUIDE) collaborative dementia care demonstration. Close to 400 care settings are or will deliver GUIDE across the U. S. over the next 7 years. The proliferation of GUIDE Model provider settings may provide opportunities for comparative effectiveness or pragmatic trials to better understand the implementation potential of dementia care innovations in real-world clinical contexts. Efforts to “crowdsource” dementia care services and supports, as in states such as Alabama, may provide an additional option for collecting more granular data on dementia caregivers and local services and supports. In crowdsourcing models, volunteers enter information about local services and supports (and themselves, potentially) through an online or mobile application that provides real-time information about dementia care programs (20, 21). Although such approaches may lack representation, they can collect local data on dementia care that underscore the implications of dementia prevalence, unmet service needs, and caregiving distress in local communities.

There are several limitations of this study. The BRFSS is administered annually by states, using a newly drawn cross-sectional sample representative of that state at each administration. Thus, analyses of within-person changes on key variables at the individual respondent level are not possible. States were selected to represent the major regions of the U. S. and exhibited strong response rates, enabling various statistical comparisons. Even within regions of the U. S., there is significant variation in culture, ethnicity, race, and socioeconomic status, meaning that the selected states remain non-representative of the broader U. S. caregiving and non-caregiving populations. For these reasons, the extent of variation within states and national samples of caregivers and non-caregivers may be greater than reported here. Another concern is whether the reported statistical differences are clinically meaningful. Although a large and representative sample is a strength of the BRFSS, even small percentage differences in variables within state or national caregiving populations may be significant for thousands of people. The measures included in the BRFSS are limited by response burden, administrative costs, and a sole reliance on self-report (including caregiver identification). Although BRFSS data are representative across states, sample sizes within states vary considerably, which may increase the risk of a Type II error, and the BRFSS may also exclude caregivers in states not captured by its survey methodology.

## 5 Conclusion

Although the advent of nationally representative data has advanced our understanding of the health implications of dementia caregiving, the local prevalence, care context, and wellbeing of dementia caregivers remain less understood. The findings of the current analysis highlight considerable variation in demographic characteristics, care contexts, and health among dementia caregivers across U. S. states. It is further likely that dementia caregiving differences at the local/community levels are even more pronounced than those observed in state and national data, as we did in this study to some extent. Utilizing localized data to its fullest extent could help advance public health efforts to elevate dementia caregiving as a priority. Nevertheless, our findings cannot account for these variations. The availability of local/community-level data, in addition to describing heterogeneity, may yield more actionable insights for local decision-makers, such as via community asset mapping (identifying local formal and informational resources to meet a community’s public health needs) to support dementia caregivers. An oft-used maxim is that all politics (and related policy) is local, and dementia care science should provide the data necessary to guide public health action for dementia caregivers in towns, communities, and similar localities as well as states.

## Data Availability

Publicly available datasets were analyzed in this study. This data can be found at: https://www.cdc.gov/brfss/data_documentation/index.htm.
